# The mediating role of adult attachment styles between early traumas and suicidal behaviour

**DOI:** 10.1038/s41598-025-00831-8

**Published:** 2025-05-06

**Authors:** Noemi Monika Szeifert, Barnabás Oláh, Xenia Gonda

**Affiliations:** 1 Doctoral School of Psychology, ELTE Eötvös Lóránd University, Budapest, Hungary; 2 Department of Sports Medicine, Semmelweis University, Budapest, Hungary; 3 Department of Behavioural Sciences, Faculty of Medicine, University of Debrecen, Debrecen, Hungary; 4 Clinical Psychology Center of CC, Health Care Service Units, University of Debrecen Clinical Centre, Debrecen, Hungary; 5 Department of Clinical Psychology, Semmelweis University, Budapest, Hungary; 6 Department of Psychiatry and Psychotherapy, Semmelweis University, Budapest, Hungary; 7 NAP3.0-SE Neuropsychopharmacology Research Group, Hungarian Brain Research Program, Semmelweis University, Budapest, Hungary

**Keywords:** Early traumas, Attachment styles, Mental disorders, Suicide attempt, Public health, Psychology, Health care

## Abstract

Our Hungarian cross-sectional study highlights the crucial mediating role that adult attachment styles play in the relationship between early traumas and suicidal behaviour, shaping how individuals process and respond to traumatic experiences. Early traumas, such as abuse or neglect, often disrupt the development of secure attachment, leading to insecure styles in adulthood—such as anxious or avoidant attachment. These insecure attachment styles influence emotional regulation, interpersonal relationships, and coping mechanisms, thereby exacerbating feelings of isolation and despair. For instance, anxious attachment can intensify fear of abandonment and hypersensitivity to rejection, increasing emotional instability and suicidal ideation. Conversely, avoidant attachment may lead to emotional suppression and reluctance to seek support, amplifying feelings of hopelessness. Psychopathological symptoms resulting from early trauma—such as depression and PTSD—are often filtered through these attachment patterns, shaping how distress is experienced and managed. In our research, we analysed the role of adult attachment styles and early traumas in suicidal behaviour. We also examined the mediating effect of attachment style in the relationship between early traumas and suicidal behaviour. A total of 357 subjects between the ages of 18 and 85 (mean = 37.02, SD = 12.86) were included in the analysis; 33.6% were male and 66.4% female. The sample consisted of 146 individuals with a history of suicide, 154 clinical participants without a history of suicide, and 57 from a non-clinical population. The adult attachment scale (AAS) and the childhood trauma questionnaire (CTQ) were used as assessment tools. To model the relationships between variables, logistic regression, generalized linear models, and mediation analyses were conducted. All models were adjusted for basic demographic variables. Our results showed that the severity of emotional abuse (adjusted OR 1.064, *p* = 0.004), emotional neglect (adjusted OR 1.064, *p* = 0.007), and overall traumatization measured by the CTQ (adjusted OR 1.021, *p* = 0.006) significantly predicted a higher risk of suicidal behaviour. In contrast, higher levels of secure attachment style predicted a lower risk of suicide attempt (adjusted OR −0.091, *p* = 0.004). Additionally, secure attachment style significantly mediated part of the total effect of early traumatization severity on suicidal behaviour (indirect effect = 0.0032, *p* < 0.05; Pm = 16.5%). We also examined the relationship between early traumas and attachment style and found multiple significant associations. For avoidant attachment, significant associations were observed with the total traumatization score (B = 0.086, *p* < 0.001) and specific adversities, including emotional abuse, emotional neglect, and physical neglect (B = 0.244–0.319, all *p* < 0.001). Anxious-ambivalent attachment was associated with the total CTQ score (B = 0.088, *p* < 0.001), as well as emotional abuse (B = 0.298, *p* < 0.001), emotional neglect (B = 0.254, *p* < 0.001), physical abuse (B = 0.248, *p* < 0.001), and physical neglect (B = 0.261, *p* = 0.010). A lower level of secure attachment was linked to the overall traumatization score (B = −0.039, *p* = 0.004), as well as emotional abuse, emotional neglect, and sexual abuse (B = −0.101 to −0.163, all *p* < 0.05). By mediating the relationship between trauma and suicidal behaviour, adult attachment styles can either perpetuate maladaptive coping strategies or hinder recovery. Understanding this mediating role is crucial for developing interventions that address attachment insecurities while promoting resilience and emotional healing. Therapeutic approaches aimed at fostering secure attachment patterns can help mitigate the effects of early trauma and reduce the risk of suicidal behaviour. These findings underscore the importance of incorporating attachment-focused strategies into trauma-informed care.

## Introduction

This complex area of research lies at the intersection of developmental psychology, mental health, and trauma studies. Attachment styles, which develop in early childhood, have a lasting influence on an individual’s mental health throughout the lifespan^1^. Secure attachment is often associated with greater resilience, whereas insecure or disorganized attachment styles can increase vulnerability to mental health disorders^2^. Traumatic experiences during formative years can disrupt typical emotional and psychological development, heightening the risk of later mental health challenges, including suicidal ideation and behaviour^3–5^. Early trauma can also shape how adult attachment styles affect suicidal behaviour. For example, individuals with high levels of early trauma may exhibit increased sensitivity to attachment-related stressors, which in turn elevates the risk of suicide^6–8^. Suicide attempt is considered a self-initiated, potentially harmful act carried out with the intent to end one’s life, which does not result in death. Such behaviour is influenced by a complex interplay of psychological, environmental, and biological factors^9^.

### Conceptual background

*Adult attachment styles* include secure, anxious, avoidant, and disorganized attachment patterns, which influence how individuals perceive and interact with others in relationships^10^. Childhood and adult attachment styles are similar in that early attachment patterns often serve as templates for adult relationships, affecting emotional bonds and interpersonal dynamics throughout life . Research suggests that the stability of attachment styles from childhood to adulthood is moderate, with about 30–40% similarity over time^11^. This percentage reflects that while early attachment experiences influence later patterns, life events and relationships can also lead to changes in attachment styles^12^. *Childhood abuse*, including emotional and physical neglect^13,14^, is strongly associated with an increased risk of suicidal behaviour^8,15,16^. According to studies on Adverse Childhood Experiences (ACEs), the lifetime prevalence of having at least one suicide attempt is 3.8%.

Experiencing adversity in any ACE category was associated with a 2- to 5-fold increased risk of attempting suicide. There was a strong, graded relationship between the total ACE score and the likelihood of suicide attempts during both childhood/adolescence and adulthood (*P* < 0.001). Compared to individuals with no ACEs—who had a 1.1% prevalence of attempted suicide—those with seven or more adverse experiences had a prevalence of 35.2% and an adjusted odds ratio of 31.1 (95% confidence interval: 20.6–47.1) for having ever attempted suicide^15^. Research has shown that adverse parental attitudes and neglect can significantly impair a child’s self-esteem and mental health, increasing vulnerability to depression and suicidal ideation^17,18^. Secure attachment to caregivers during early development plays a crucial role in shaping emotional regulation, stress response systems, and social functioning later in life^1^. In contrast, disruptions in these early bonds—particularly the formation of insecure or disorganized attachment styles—are associated with elevated risks for anxiety, depression, and suicidal ideation in both adolescence and adulthood^19,20^. These early attachment patterns contribute to the development of internal working models that influence how individuals perceive themselves and others in relationships throughout life^21^. Additionally, it is important to consider the various theoretical models used to conceptualize adult attachment, such as Bartholomew and Horowitz’s (1991) four-category model^22^, which distinguishes between secure, fearful, preoccupied, and dismissing attachment styles. These models offer more nuanced insights into the ways attachment functions in adulthood and highlight differences in relational expectations and self-other perceptions. However, the lack of consensus around which model to apply can impact how research findings are interpreted and compared across studies. This variability underscores the importance of clearly defining the attachment framework used in empirical investigations, especially when examining outcomes like suicidal behaviour. Furthermore, children born from unplanned pregnancies are at higher risk of developing insecure attachments and experiencing emotional neglect and behavioural challenges—factors that further elevate the likelihood of suicidal behaviour later in life^15,23^. The *Adverse Childhood Experiences* (ACE) Study reinforces these findings, demonstrating a strong, graded relationship between exposure to childhood trauma—including neglect and household dysfunction—and the lifetime risk of suicide attempts^17^. Individuals who have endured complex trauma (chronic, multiple traumatic experiences often beginning in early childhood) are at higher risk for suicidal thoughts and attempts due to the profound impacts on attachment, self-concept, and emotional stability^24^. These traumatic experiences often lead to the development of psychopathological symptoms like depression, anxiety, posttraumatic disorder (PTSD) or Complex PTSD (CPTSD), which further exacerbate suicidal ideation. The interplay between unresolved trauma, insecure attachment styles and psychopathological symptoms creates a cycle of psychological distress and increasing the risk of suicide.

Research has demonstrated that adult attachment styles, particularly insecure attachments, and early traumas are significantly associated with suicidal behaviour^8,25^. These studies highlight the significant impact of both attachment insecurity and early trauma on the risk for suicidal behaviour^26^, with attachment styles often mediating this relationship. For instance, insecure attachment styles developed due to early trauma can lead to poor emotional regulation, interpersonal challenges, and feelings of hopelessness, all of which increase the risk of suicidality^27^. Avoidant individuals tend to suppress or avoid expressing their emotions, which can lead to accumulated emotional distress. This avoidance of seeking support or expressing vulnerability has been linked to an increased risk of suicidal behaviour as they may feel isolated or unable to manage distressing thoughts alone^28^. This emotional suppression can create an internalized build-up of distress that, without release or external help, may drive them to view suicide as an escape from these intense, unmanageable emotions^19,29^. Disorganized attachment is marked by a contradictory mix of seeking closeness and feeling fearful or ambivalent toward relationships. This attachment style reflects the confusion and lack of coherent coping strategies that result when caregivers are both a source of fear and comfort^30,31^. It is often associated with the highest risk of suicide due to chaotic relational patterns and internalized trauma^32^. Those with secure attachments typically have better coping mechanisms, resilience, and social support, which can act as protective factors against suicidality even in the presence of trauma, whereas those with insecure attachments may lack these protective factors^19^. Attachment styles may influence how individuals cope with early trauma, psychopathological symptoms (e.g. personality disorders, alcohol dependency, substance abuse), harmful life management and, consequently, their risk of engaging in suicidal behaviour.

Early trauma and attachment insecurity can result in lasting changes in the brain’s stress regulation systems, such as the hypothalamic-pituitary-adrenal (HPA) axis, leading to heightened vulnerability to stress and emotional dysregulation later in life. This dysregulation is a key factor in the pathogenesis of suicidal behaviour^33^. Studies on brain imaging also show altered brain connectivity and functioning in areas related to attachment, emotion processing, and regulation in individuals who have experienced early trauma, which may contribute to their increased risk for suicide^34^.

Our research examines how adult attachment styles mediate the relationship between early traumatic experiences and suicidal behaviour. The study may reveal that certain combinations of attachment style and trauma increase suicide risk, informing targeted interventions. A sample that includes individuals with varying attachment styles and trauma histories could offer insights into different moderating effects.

## Research methodology

### Measures

Demographic data were collected through self-report questionnaires and supplemented with information from the hospital’s electronic system, following the patients’ informed consent. The response categories of some demographic variables were combined, resulting in the following categorisation: education: primary, secondary, tertiary; marital status: living alone, living in any type of relationship; financial status: below average, average, above average.

The *adult attachment scale (AAS)* is a self-report measure developed to assess attachment patterns in adults, specifically focusing on three dimensions: closeness, dependency, and anxiety. Each dimension reflects core aspects of attachment, with closeness and dependency measuring comfort with intimacy and reliance on others, while anxiety assesses fear of rejection or abandonment. The AAS has demonstrated good internal consistency, with Cronbach’s alpha values generally ranging between 0.70 and 0.80^35^. It also has strong construct validity, correlating well with related attachment and interpersonal measures, making it a widely used tool in research on adult attachment. The Hungarian adaptation shows Cronbach α = 0.306 and 0.705^36^.

The *childhood trauma questionnaire* (CTQ) is a widely used self-report instrument designed to assess the severity and frequency of childhood abuse and neglect across five domains: emotional abuse, physical abuse, sexual abuse, emotional neglect, and physical neglect. It consists of 28 items rated on a Likert scale, ranging from 1 (never true) to 5 (very often true) providing both a total trauma score and individual scores for each trauma type. The CTQ demonstrates high internal consistency, with Cronbach’s alpha values typically ranging from 0.70 to 0.95 across subscales, indicating strong reliability^37^. The internal reliability for CTQ in the current sample varies Cronbach α = 0.0630–0.914. The Hungarian adaptation was processed by Csernela et al., 2021^38^.

*Suicide attempts* were assessed using a combination of self-report questionnaires—where participants (inpatients) were asked If they had a history of suicide attempts—and structured clinical interviews conducted by psychiatrists and trained clinical psychologists. Cases classified as parasuicidal behaviour (non-suicidal self-injury or gestures without intent to die) were excluded from the sample.

### Participants

Participants in the patient group were recruited from the Péterfy Sándor Hospital, Crisis Intervention and Psychiatric Ward, Budapest, Hungary. Psychiatric diagnoses were based on the Structured Clinical Interview for DSM-IV and ICD-10 administered by psychiatrists and trained clinical psychologists. Subjects were excluded with acute psychotic episodes, current alcohol or substance abuse, or schizophrenia, history of traumatic brain injury or neurological disorder, or serious medical condition. Healthy control participants were recruited from the Hungarian University of Sports Science, subjects who neither have ever been under psychiatric treatment nor have suicide history. All subjects were involved in the study on a voluntary basis and all of them signed a written informed consent. This study was approved by both Institutional Review Boards, IRB of Péterfy Sándor Hospital and Outpatient Center (approval number 25/2016) and IRB of Hungarian University of Sports Science (approval number MTSE-OKE-KEB/04/2023). The authors assert that all procedures contributing to this work comply with the ethical standards of the relevant national and institutional committees on human experimentation and with the Helsinki Declaration of 1975, as revised in 2013. After signing a written informed consent form, the AAS and CTQ were administered to the involved subjects as paper-pencil tests. 357 subjects were administered to fill out the inventories, 300 clinical and 57 non-clinical subjects, between the ages of 18–85 (mean 37.02; SD 12.86), 33.61% male, 66.39% female. The sample distribution is in line with the literature and clinical experience that women are more represented in psychiatric settings. The demographic characteristics of the sample are listed in (Table 1). The frequency of the diagnoses in the clinical group are follows: Anxiety disorders (44.54%), Major depressive disorder (39.22%), Bipolar disorder (14.01%), Borderline Personality Disorder (24.93%), Personality disorder not specified (22.13%), non-clinical sample (15.97%). As a comorbidity in the clinical group, the following addictions occurred: alcohol dependence (25.49%), substance dependence (12.61%), drug dependency (benzodiazepine most frequently) (20.17%), and nicotine dependency (40.46%). 40.89% of the patients have at least one suicide attempt in their history (27,4% male, 72,6% female). This study does not provide a detailed analysis of gender differences for the research questions due to the unequal distribution of genders. The role of diagnosis in suicide attempts was not examined in this study either, since it has already been published in a previous paper.

## Statistical analyses

Statistical analyses were conducted using IBM SPSS Statistics v.23 (IBM Corporation, Armonk, NY, USA). Secure attachment followed a normal distribution, while the other two styles showed slight deviations (Shapiro-Wilk test). Other continuous variables were non-normally distributed. Demographics were summarized using means, standard deviations, medians, interquartile ranges, or proportions for the full sample and subsamples by suicidal behavior history.

The Mann-Whitney U test and Pearson’s chi-squared test assessed unadjusted associations between early trauma, attachment styles, and suicidal behavior. Generalized linear models examined the relationship between traumatization and attachment. Logistic regression predicted suicide risk separately for traumatization and attachment. All models were adjusted for adjusted for basic demographics (gender, age, location, education, marital and financial status). Response categories of some demographic variables were combined to improve the interpretability, the stability and efficiency of the models. Post-test analysis used the adjusted Wald test.

Mediation analysis, performed with Hayes Process Macro v.4.0 (Hayes, 2013), tested whether attachment style mediated the link between early trauma and suicidal behavior.

## Results

### The study samples

The demographic characteristics of the samples overall and by history of suicidal behaviour are shown in (Table 1). Significant demographic differences were found in gender, education, marital status and financial status, whereas age and place of residence did not differ significantly between groups. Regarding gender, a significantly higher proportion of females (73.5%) reported suicidal behaviour compared to males (26.5%) (χ²(1) = 5.023, *p* = 0.025). Educational level also varied, with those with a history of suicidal behaviour being about half as likely to have a university education (26.5%) compared to those without a history of suicidal behaviour (50.7%) (χ²(3) = 20.403, *p* < 0.001). In terms of marital status, single people were more common in the suicide attempters (45.6%) than in the non-suicidal group (31.2%) (χ²(4) = 14.823, *p* = 0.005). Subjective financial status showed further differences, with a higher proportion of those with suicidal behaviour reporting poor financial status (20.6%) compared with the no-attempt group (9.5%), while good financial status was more common among those without suicidal behaviour (27.6%) (χ²(4) = 11.671, *p* = 0.020). Non-significant differences were found in age (with a mean age of around 37 years in both groups) and place of residence, with similar distributions across capital city, regional city, rural town and village.

**Table 1:** Demographic characteristics of the samples overall and by the history of suicidal behaviour.

	Total sample (*n*=357)	Suicidal behaviour (*n*=146)	No suicidal behaviour (*n*=211)	Test statistics^i^
Gender *n* (%)				
Male	120 (33.6%)	36 (26.5%)	84 (38%)	χ²(1) = 5.023, *p* = 0.025
Female	237 (66.4%)	100 (73.5%)	137 (62%)	
Age mean (SD)/mdn (IQR)	37.02 (12.86)/35.0 (26–46)	36.85 (12.56)/35.5 (26–45.75)	37.13 (13.08)/35.0 (26–48)	U = 14,922, *p* = 0.705
Residence n (%)				
Capital city	199 (55.7%)	77 (56.6%)	122 (55.2%)	χ²(3) = 1.834, *p* = 0.607
Regional city	44 (12.3%)	13 (9.6%)	31 (14%)	
Rural town	77 (21.6%)	30 (22.1%)	47 (21.3%)	
Village	37 (10.4%)	16 (11.8%)	21 (9.5%)	
Educational level n (%)				
Primary	26 (7.3%)	13 (9.6%)	13 (5.9%)	χ²(3) = 20.403, *p* < 0.001
Vocational	56 (15.7%)	27 (19.9%)	29 (13.1%)	
High school	127 (35.6%)	60 (44.1%)	67 (30.3%)	
Higher education	148 (41.5%)	36 (26.5%)	112 (50.7%)	
Marital status n (%)				
Single	131 (36.7%)	62 (45.6%)	69 (31.2%)	χ²(4) = 14.823, *p* = 0.005
In relationship	84 (23.5%)	22 (16.2%)	62 (28.1%)	
Married/cohabiting	107 (30.0%)	37 (27.2%)	70 (31.7%)	
Divorced	24 (6.7%)	13 (9.6%)	11 (5.0%)	
Widowed	11 (3.1%)	2 (1.5%)	9 (4.1%)	
Subjective financial status n (%)				
Very poor	22 (6.2%)	8 (5.9%)	14 (6.3%)	χ²(4) = 11.671, *p* = 0.020
Poor	49 (13.7%)	28 (20.6%)	21 (9.5%)	
Moderate	192 (53.8%)	73 (53.7%)	119 (53.8%)	
Good	84 (23.5%)	23 (16.9%)	61 (27.6%)	
Excellent	10 (2.8%)	4 (2.9%)	6 (2.7%)	

^i^Indicates the application of Pearson’s chi-squared test or Mann-Whitney U test.

### Early traumatic experiences and attachment styles in the samples

Table 2 provides a descriptive overview of early traumatic experiences and attachment styles within the sample, with unadjusted analyses of differences between groups.

Childhood trauma questionnaire (CTQ) scores illustrate differences in adversity, with a median CTQ score of 45.00 (IQR 33.00–56.00) across the sample. The group of those who attempted suicide scored higher, with a median of 49.00 (IQR 36.00–63.00), while non-attempters had a median of 42.00 (IQR 31.00–52.00). They also scored higher on specific early traumatic experiences measured by the CTQ, with medians of 14.00 for emotional abuse compared to 11.00 for non-attempters, 13.00 for emotional neglect compared to 10.00, 8.00 for physical abuse compared to 6.00, and 6.00 for physical neglect compared to 5.00 for non-attempters. Sexual abuse scores had similar medians (5.00), but with a wider range for attempters. Medians for attachment style show different patterns between suicide attempters and non-attempters. Secure attachment had a median score of 10.00 (IQR 7.00–12.00) for those with a history of suicidal behaviour, slightly lower than the median of 11.00 (IQR 9.00–14.00) for non-attempters. Avoidant attachment had a median of 11.00 (IQR 7.00–16.00) for attempters, slightly higher than the median of 10.00 (IQR 6.00–14.00) for non-attempters. For anxious/ambivalent attachment, the median score for attempters was 12.00 (IQR 8.00–17.00), compared with a median of 10.00 (IQR 5.00–16.00) for non-attempters. This suggests a trend towards greater avoidant and anxious-ambivalent attachment tendencies in those with a history of suicidal behaviour. However, the statistical associations shown in Table 2 are not adjusted for confounding factors. Adjustments were made in subsequent analyses.

**Table 2:** Descriptive statistics of early traumatic experiences and attachment styles in the samples and their unadjusted associations with suicidal behaviour.

	Total sample (*n*=357)	Attempted suicide (*n*=146)	No suicidal behaviour (*n*=211)	Test statistics^i^
Childhood trauma questionnaire (CTQ)				
CTQ total Mdn (IQR)	45.00 (33.00–56.00)	49.00 (36.00–63.00)	42.00 (31.00–52.00)	U = 9782.0, *p* < 0.001
Emotional abuse Mdn (IQR)	12.00 (8.00–17.00)	14.00 (9.00–18.00)	11.00 (7.00–16.00)	U = 10814.5, *p* < 0.001
Emotional neglect Mdn (IQR)	11.00 (6.00–16.00)	13.00 (7.00–18.00)	10.00 (5.00–14.00)	U = 11001.0, *p* < 0.001
Physical abuse Mdn (IQR)	7.00 (5.00–9.00)	8.00 (6.00–10.00)	6.00 (5.00–9.00)	U = 11417.5, *p* = 0.018
Physical neglect Mdn (IQR)	6.00 (5.00–8.00)	6.00 (5.00–10.00)	5.00 (5.00–7.00)	U = 11574.5, *p* = 0.008
Sexual abuse Mdn (IQR)	5.00 (5.00–8.00)	5.00 (5.00–8.00)	5.00 (5.00–7.00)	U = 11799.0, *p* = 0.089
Attachment style				
Secure Mdn (IQR)	11.00 (8.00–13.00)	10.00 (7.00–12.00)	11.00 (9.00–14.00)	U = 12011.5, *p* = 0.248
Avoidant Mdn (IQR)	11.00 (7.00–15.00)	11.00 (7.00–16.00)	10.00 (6.00–14.00)	U = 11443.0, *p* = 0.013
Anxious-ambivalent Mdn (IQR)	11.00 (6.00–17.00)	12.00 (8.00–17.00)	10.00 (5.00–16.00)	U = 11232.5, *p* = 0.032

^i^Indicates the application of Pearson’s chi-squared test or Mann-Whitney U test.

**Table 3:** Associations of early traumatic experiences with attachment styles.

	Attachment style					
	Secure		Avoidant		Anxious-ambivalent	
	B^i^	*p*-value	B^i^	*p*-value	B^i^	*p*-value
CTQ total	−0.039	0.004*	0.086	<0.001*	0.088	<0.001*
Emotional abuse	−0.101	0.011*	0.244	<0.001*	0.298	<0.001*
Emotional neglect	−0.119	0.003*	0.319	<0.001*	0.254	<0.001*
Physical abuse	−0.068	0.898	0.165	0.055	0.248	0.008*
Physical neglect	−0.099	0.149	0.294	<0.001*	0.261	0.010*
Sexual abuse	−0.163	0.017*	0.103	<0.265	0.074	0.465

^i^Generalized linear models, adjusted for gender, age, location, education, marital status and subjective financial status.

*The model including the coefficient is significant at a level of *p*<0.05.

Attachment styles were predicted by a wide range of early traumatic experiences in the sample after controlling for basic demographics. In Table 3 the B-values from the generalized linear models represent the strength and direction of the association between the trauma variables and each attachment style. A positive B-value indicates that a greater severity of early trauma is associated with higher levels of that attachment style, while a negative B-value indicates the opposite. For avoidant attachment, significant associations were observed with total trauma score (B = 0.866, *p* < 0. 001) and specific adversities including emotional abuse (B = 0.244, *p* < 0.001), emotional neglect (B = 0.319, *p* < 0.001), physical neglect (B = 0.294, *p* < 0.001) and with physical abuse showing marginal significance (B = 0.165, *p* = 0.055). Anxious-ambivalent attachment showed significant associations with CTQ total score (B = 0.088, *p* < 0.001), emotional abuse (B = 0.298, *p* < 0.001), emotional neglect (B = 0.254, *p* < 0.001), physical abuse (B = 0.248, *p* = 0.008) and physical neglect (B = 0.261, *p* = 0.010). The level of secure attachment was negatively associated with total CTQ score (B=−0.039, *p* = 0.004), emotional abuse (B=-0.101, *p* = 0.011), emotional neglect (−0.119, *p* = 0.003) and sexual abuse (B=−0.163, *p* = 0.017).

### The relationship of early traumatic experiences and attachment styles with suicidal behaviour

We modelled the relationship between early traumatic experiences and the likelihood of suicidal behaviour by using logistic regression models adjusted for basic demographics (Table 4). The overall severity of traumatisation score on the CTQ showed a significant association with the risk of suicidal behaviour, meaning that for every one-point increase in the cumulative CTQ score, the odds of suicidal behaviour increased by 2.1% (OR = 1.021, *p* = 0.006). Within the CTQ subscales, both emotional abuse (OR 1.064, *p* = 0.004) and emotional neglect (adjusted OR 1.064, *p* = 0.007) emerged as significant predictors of suicidal behaviour. Each additional point in the emotional abuse or neglect score was associated with a 6.4% increase in the odds of suicidal behaviour.

**Table 4:** Modelling the associations of early traumatic experiences and suicidal behaviour.

	Suicidal behaviour	
	OR^i^	*p*-value
Total CTQ	1.021	0.006*
Emotional abuse	1.064	0.004*
Emotional neglect	1.064	0.007*
Physical abuse	1.104	0.159
Physical neglect	1.063	0.103
Sexual abuse	1.060	0.112

^i^Logistic regression models with entry method, adjusted for gender, age, location, education, marital status and subjective financial status.

*The model including the coefficient is significant at a level of *p*<0.05.

When we modelled the associations between attachment styles and suicidal behaviour by calculating adjusted odds ratios (Table 5), secure attachment showed a significant negative association with suicidal behaviour (OR −0.091, *p* = 0.004), meaning that a one-point increase in secure attachment was associated with a 91% reduction in the odds of suicidal behaviour (Table 5). In contrast, avoidant attachment (OR 0.028, *p* = 0.233) and anxious-ambivalent attachment (OR 0.040, *p* = 0.059) did not reach statistical significance in predicting suicidal behaviours.

**Table 5:** Modelling the associations of attachment styles and suicidal behaviour.

Attachment style	Suicidal behaviour	
	OR^i^	*p*-value
Secure	−0.091	0.004*
Avoidant	0.028	0.233
Anxious-ambivalent	0.040	0.059

^i^Logistic regression models with entry method, adjusted for gender, age, location, education, marital status and subjective financial status.

*The model including the coefficient is significant at a level of *p*<0.05.

### Analysing the mediating effect of attachment styles between early traumatic experiences and suicidal behaviour

**Fig. 1:**
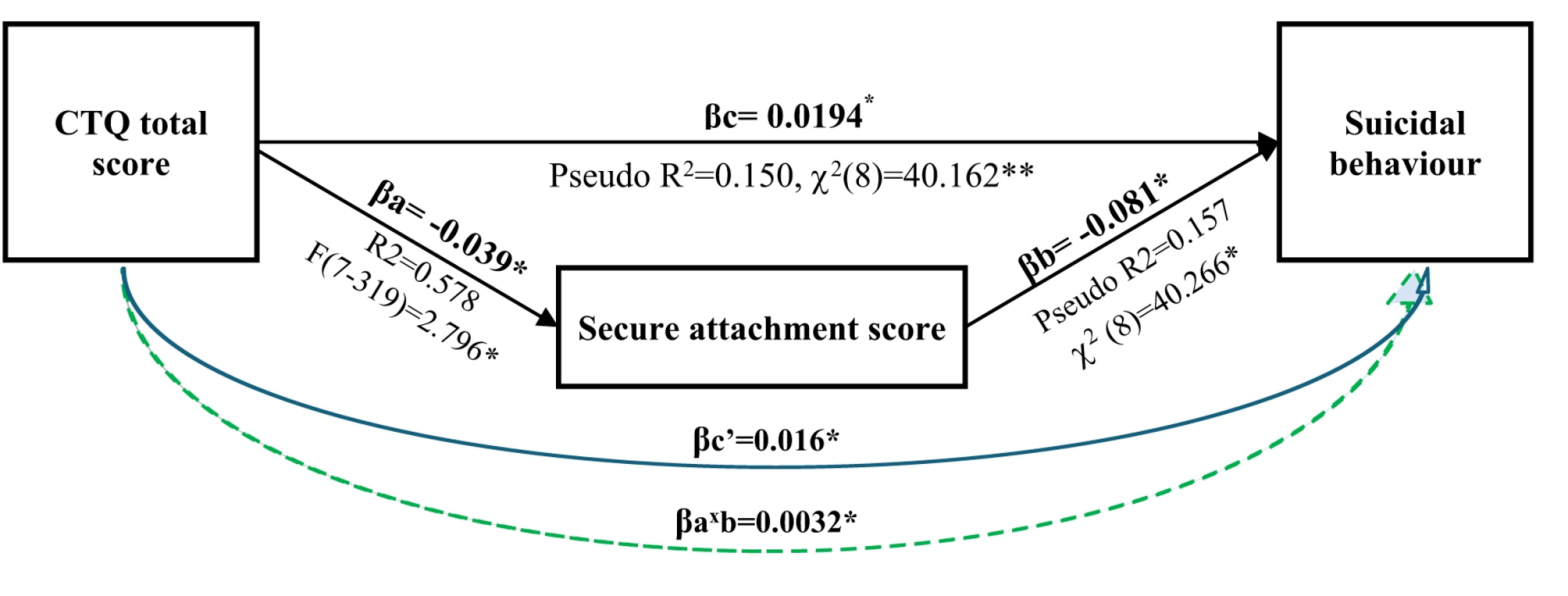
Mediating role of secure attachment in the relationship between early traumatic experiences and suicidal behaviour. Percentage of mediation (Pm) = 0.165. **p < 0.01, *p < 0.05.

We conducted a mediation analysis that highlighted the mediating role of secure attachment style between the severity of early traumatic experiences (as measured by the CTQ) and risk of suicidal behaviour (Fig. 1). Our results indicated significant pathways. The overall effect of CTQ total score on suicidal behaviour was positive (βc = 0.0194, *p* < 0.05), suggesting that more severe exposure to early traumatic experiences was associated with an increased likelihood of suicidal behaviour. When secure attachment was entered as a mediator, the direct effect of early traumatic experiences (βc′ = 0.016, *p* < 0.05) was reduced from the unmediated path (path c) and its indirect effect (βa × βb = 0.0032, *p* < 0.05) was significant. The result indicated that part of the effect of early traumatic experiences on suicide risk was mediated by secure attachment levels. The regression model achieved a pseudo-R² of 0.157 (χ²(8) = 40.266, *p* < 0.05), reflecting a modest but statistically significant fit. The proportion of mediation (Pm) was 16.5%, meaning that reduced secure attachment accounted for 16.5% of the total effect of early traumatic experiences on suicide risk.

## Discussion

Our findings demonstrate that elevated levels of early childhood trauma and insecure attachment styles are significantly associated with an increased risk of suicidal behaviour. Insecure attachment styles not only intensify the detrimental psychological effects of childhood maltreatment but also contribute to persistent emotional vulnerability, characterized by difficulty forming stable emotional bonds, heightened mistrust, and impaired affect regulation^10,39^. This emotional fragility, often rooted in early relational trauma, may result in pervasive feelings of abandonment and low self-worth, ultimately fostering maladaptive coping mechanisms such as self-harm or suicidal ideation. Avoidant attachment tendencies may drive individuals to suppress emotional needs and resist seeking support, while anxious attachment may exacerbate dependency and fear of rejection—both dynamics increasing suicide risk^40^. Furthermore, insecure attachment undermines resilience, impedes therapeutic engagement, and is frequently accompanied by psychopathological symptoms such as depression, anxiety, and emotional dysregulation, all of which amplify vulnerability to self-destructive behaviours^21,41^. Consistent with these theoretical models, our results revealed that the severity of emotional abuse (adjusted OR = 1.064, *p* = 0.004), emotional neglect (adjusted OR 1.064, *p* = 0.007), and cumulative childhood trauma (CTQ total score; adjusted OR 1.021, *p* = 0.006) significantly predicted increased suicidal behaviour. In contrast, secure attachment served as a protective factor (adjusted OR −0.091, *p* = 0.004) and partially mediated the relationship between trauma severity and suicidal risk (indirect effect = 0.0032, *p* < 0.05; Pm = 16.5%), suggesting its role as a psychological buffer in the face of adversity (Taylor et al., 2015). Additional analyses revealed that avoidant attachment was significantly associated with emotional abuse, emotional neglect, and physical neglect (B = 0.244–0.319, all *p* < 0.001), while anxious-ambivalent attachment correlated with a broader range of adverse experiences, including emotional and physical abuse, neglect, and overall CTQ scores (B = 0.088–0.298, all *p* < 0.001). Conversely, lower levels of secure attachment were significantly linked with greater exposure to emotional abuse, emotional neglect, and sexual abuse (B = −0.101 to −0.163, all *p* < 0.05). These findings align with existing literature underscoring the long-term impact of early interpersonal trauma on attachment systems and psychological functioning^42,43^, reinforcing the importance of incorporating attachment-based approaches in interventions aimed at mitigating suicide risk among individuals with histories of childhood maltreatment.

## Strengths and limitations

Limitations of this study include its cross-sectional design, which limits causal interpretations, and its reliance on self-reported data of sensitive topics such as childhood trauma and attachment, which may introduce biases such as recall bias. In addition, the specific demographic characteristics of the sample and the lack of representativeness limit the generalisability of the findings. Although the study uses established measure (CTQ), it may not capture the full complexity of traumatic experiences. On the other hand, the study has several strengths. The use of validated instrument enhances measurement reliability, while the Hayes Process macro supports a rigorous examination of mediation effects. The adjustment for demographic variables strengthens the internal validity of the study by reducing the likelihood that the results are confounded by these factors. Additional limitations of this study include the absence of assessments for trauma-related symptoms such as Post-Traumatic Stress Disorder (PTSD) or complex PTSD (CPTSD), which may have influenced the observed associations. Furthermore, the lack of a general population comparison or control group limits the ability to contextualize the findings within a broader population. Finally, the study did not explore whether the mediational pathways linking adverse childhood experiences to mental health outcomes vary according to the specific type of trauma experienced, which could provide important insights for targeted interventions.

## Conclusions

Understanding the link between insecure attachment and the effects of childhood maltreatment is crucial for developing targeted, trauma-informed treatments. Interventions that address attachment insecurities may reduce the likelihood of suicide attempts and promote emotional healing. Addressing these interconnected factors through trauma-informed care and attachment-focused interventions is essential to break this cycle and reduce the risk of suicide attempts. This study could significantly contribute to understanding how attachment styles and early traumas interact to influence suicidal behaviour. Addressing these factors in treatment can lead to personalized and effective interventions, potentially reducing suicide risk in vulnerable individuals. Mental health professionals might focus on fostering secure attachments in therapy or directly addressing trauma-related issues to mitigate suicidal tendencies. Insights could aid in developing prevention strategies for at-risk populations, particularly those with insecure attachment patterns and high levels of early trauma. Promoting attachment security is crucial in preventing mental health issues, as early secure attachments provide a foundation for emotional regulation, resilience, and healthy interpersonal relationships. Insecure or disrupted attachments are linked to a higher risk of depression, anxiety, and suicidal behaviours later in life. Integrating attachment-focused interventions—such as parent-child therapy, trauma-informed care, and relationship-based support—into mental health promotion efforts can help strengthen emotional bonds, enhance coping skills, and reduce the long-term psychological impact of early adversity. These interventions can be especially effective when implemented early in life or during key developmental transitions.

## Data Availability

Data available upon reasonable request by Noemi Szeifert (e-mail: szeifert.noemi@semmelweis.hu).
